# State-of-Art in the Age Determination of Venous Thromboembolism: A Systematic Review

**DOI:** 10.3390/diagnostics11122397

**Published:** 2021-12-20

**Authors:** Nicola Di Fazio, Giuseppe Delogu, Costantino Ciallella, Martina Padovano, Federica Spadazzi, Paola Frati, Vittorio Fineschi

**Affiliations:** 1 Department of Anatomical, Histological, Forensic and Orthopedic Science, Sapienza University of Rome, 00161 Rome, Italy; nicola.difazio@uniroma1.it (N.D.F.); giuseppe.delogu@uniroma1.it (G.D.); costantino.ciallella@uniroma1.it (C.C.); martina.padovano@uniroma1.it (M.P.); federica.spadazzi@uniroma1.it (F.S.); paola.frati@uniroma1.it (P.F.); 2 Istituto di Ricovero e Cura a Carattere Scientifico (IRCCS) Neuromed, Via Atinense 18, 86077 Pozzilli, Italy

**Keywords:** forensic pathology, immunohistochemistry, thromboembolism, thrombus age determination, neutrophils, macrophages, fibrocyte, myofibroblast, neo-vessels

## Abstract

Venous thromboembolism (VTE), consisting of deep vein thrombosis (DVT) and pulmonary embolism (PE), requires a forensic age determination to ascertain their causal relationship with recent events, such as trauma or medical treatment. The main objective of this systematic review is to identify the current state-of-the-art immunohistochemical methods for age determination of fatal VTE. A literature search was performed through different databases, according to Preferred Reporting Items for Systematic Reviews and Meta-Analyses (PRISMA) guidelines. Within the study, we have selected only cases represented by deceased patients for DVT and/or PTE in which thromboembolic material was collected during an autoptic examination and then subjected to a histological and an immunohistochemical investigation. Studies based on animal models were not included. We assessed bias risk. A database-based search produced a total of 19 articles. After excluding duplicate items from the selection, 14 articles were reviewed. Ten articles were excluded because they did not meet the inclusion criteria. The results have pointed out 4 studies that were included in the present analysis for a total of 157 samples of DVT and 171 PTE samples. These were analyzed using traditional histological and immunohistochemical techniques. The results must be interpreted with a critical eye because of their heterogeneity in terms of time, geography, and study design. The present review highlights the importance of associating specific immunohistochemical markers with a histological analysis for the timing of DVT/PTE fatal events. Further future experiences will hopefully endorse actual knowledge on the subject to increase the accuracy in the assessment of thrombus-embolus age.

## 1. Introduction

Venous thromboembolism (VTE) consists of two clinical conditions: deep vein thrombosis (DVT) and pulmonary embolism (PE). They certainly share the same pathogenesis, but the way they develop may vary from clinically silent, to massive embolism causing death [1,2].

Acute PE is the most serious clinical presentation of VTE. Since PE is, in most cases, the consequence of DVT, most of the existing data on its epidemiology, risk factors, and natural history are derived from studies that have examined VTE as a whole [3,4]. The epidemiology of PE is difficult to determine because it may remain asymptomatic, or its diagnosis may be an incidental finding; [5] in some cases, as we have already explained, the first presentation of PE may be sudden death [6,7].

VTE is the third most frequent cardiovascular disease with a total annual incidence ranging from 104 to 183 cases per 100,000 population [4,5,8]. The total incidence seems to be similar to that of brain stroke and is more common in Afro-Americans, while it is less common in Asians and Native Americans. It is a disease that mainly affects old age and is rare before late adolescence. The incidence increases with age in both sexes and is higher in men (130 cases per 100,000 population) than in women (110 cases per 100,000 population).

### 1.1. Pathophysiology

VTE is considered to be a consequence of the interaction between patient-related (usually permanent) [9] risks factors and setting-related (usually temporary) risk factors [10]. Virchow’s triad summarizes the factors that predispose to thrombus formation: endothelial dysfunction, turbulence or stasis of blood flow, and a hypercoagulability state. Therefore, the acquired and hereditary factors for thrombophilia contribute to the onset of this multifactorial pathology [11]. VTE is ‘provoked’ in the presence of a temporary or reversible risk factor (such as surgery, trauma, immobilization, pregnancy, oral contraceptive use, or hormone replacement therapy) within the last 6 weeks to 3 months before a diagnosis [3], and ‘unprovoked’ in the absence thereof. Major trauma, surgery, lower limb fractures, joint replacements, and spinal cord injury, are strong provoking factors for VTE. Cancer is a well-recognized predisposing factor for VTE.

The risk of VTE varies with different types of cancer; [6] hematological malignancies, lung cancer, gastrointestinal cancer, pancreatic cancer, and brain cancer carry the highest risk [2,11,12,13]. Among fertile women, oral contraception is the most frequent predisposing factor for VTE [14,15]. In vitro fertilization further increases the risk of pregnancy-associated VTE [16]. In post-menopausal women who receive hormone replacement therapy, the risk of VTE varies widely depending on the formulation used [17]. An infection is a common trigger for hospitalization for VTE [4,18]. Blood transfusion and erythropoiesis-stimulating agents are also associated with an increased risk of VTE.

Serious chronic medical conditions [19] and central venous lines are considered to be likely triggers of PE. VTE may be viewed as part of the cardiovascular disease continuum and common risk factors (such as cigarette smoking, obesity, hypercholesterolemia, hypertension, and diabetes mellitus) are shared with arterial disease, notably atherosclerosis. However, at least in part, this may be an indirect association, mediated by the effects of coronary artery disease and, in the case of smoking, cancer. Myocardial infarction and heart failure increase the risk of PE; conversely, patients with VTE have an increased risk of subsequent myocardial infarction and stroke [2,11,12].

### 1.2. Clinical Presentation

Deep vein thrombosis (DVT) of the lower extremity is usually categorized into three types: iliac, femoral, and crural types. Although the crural type does not cause clinical symptoms, it was found to have a higher risk of proximal propagation causing pulmonary embolism. Among the crural veins, the highest detection rate of thrombi was detected in the soleal vein [20]. The clinical diagnosis of deep vein thrombosis and pulmonary embolism has a sensitivity between 10% and 30%, while from autoptic cases it emerges that only 15–45% of cases of fatal pulmonary thromboembolism are recognized before death. With such low sensitivity, the autopsy assessment is confirmed to be the best examination for diagnosing pulmonary thromboembolism [21,22,23].

The lack of specificity of the symptoms and the fact that the pulmonary embolism can arise both in healthy subjects and in patients suffering from other pathologies explains the difficulty of making the correct diagnosis [24]. As a result, these diagnostic errors lead to frequent complaints, as well as to medical negligence litigation of the health authorities intervening in individual cases.

### 1.3. Relevance in the Forensic Setting

In the forensic and medico-legal setting, the importance of PTE is performed by two main items [25]. First, it is important to establish the causal relationship between PTE and recent events, such as trauma or medical treatment. Concerning medico-legal liability, the following crucial question should be considered: Was this lethal PTE an unpreventable complication or was it the consequence of real medical malpractice? As it concerns PTE, medical malpractice can theoretically occur as a misdiagnosis of deep venous thrombosis (DVT) or as a lack of the correct prophylaxis in selected patients [26]. Focusing on DVT-PTE misdiagnosis, the limit between simple complication and true medical malpractice is often blurred: less than 25% of patients having acute DVT-PTE show typical symptoms (dyspnea, chest pain, and hemoptysis), early stages of DVT-PTE can be completely asymptomatic, and up to 25% of patients with acute PTE suddenly die without an appreciable clinical syndrome. In the clinical setting, just 33% of the PTE cases are successfully diagnosed, and PTE is estimated to be the terminal cause of death for 5–10% of all the inpatients. Independent autopsy series agree that only 15–45% of lethal PTE cases are successfully recognized by clinicians before death occurred [9,10,27].

Finally, in the case of sudden cardiovascular death, investigations need to be carried out, as PTE is one of the most frequently missed diagnoses in sudden, unexpected death.

Thus, the rationale of the age determination of the DVT and PTE onset time, which represents a challenge for the forensic pathologist, is to establish the causal relationship between patients’ predisposing factors, medical treatment, thromboembolic event, and death.

### 1.4. Objectives of the Study

It therefore follows that, in the forensic context, the determination of the time of onset of thrombosis is an element of primary importance [28,29,30]. In the past, models based on the histological investigation have been proposed, such as that of Irninger in 1963 [31] and that of Fineschi et al. in 2009 [30]. However, the development of new methods, with greater sensitivity and specificity using time-specific anti-protein antibodies, allows for obtaining a greater diagnostic accuracy in the delicate task of establishing the timing of fatal pulmonary thromboembolism, especially in cases related to medical professional responsibility. Therefore, this work is necessitated by the current lack of a systematic review aimed at summarizing the scientific evidence acquired so far on post-mortem pulmonary thromboembolism dating through immunohistochemical investigations.

The main objective of this systematic review is to identify the current state-of-the-art immunohistochemical methods for the evaluation of the age of fatal pulmonary thromboembolism. Secondly, based on the knowledge acquired so far, it is intended to correlate the available data for constructing a diagnostic algorithm.

## 2. Materials and Methods

The present systematic review was prepared according to the Preferred Reporting Items for Systematic Reviews and Meta-Analyses (PRISMA) standards [32]. The focused question of the present study was: “What is the current gold standard about the timing of fatal pulmonary thromboembolism in cases subjected to autopsy?”.

### 2.1. Inclusion Criteria

We selected the study to be included in the present systematic review based on the following characteristics:

- Patients: bodies subjected to autopsy with evidence of deep vein thrombosis (DVT) and/or pulmonary arterial thromboembolism (PTE);
- Intervention: autoptic thromboembolic material sampling and immunohistochemistry investigation for age determination;
- Comparison: Fineschi et al. classification based on histological method;
- Outcome: accuracy of method and indicators used, correspondence with histological results, concordance of different studies on pulmonary thromboembolism dating.

### 2.2. Exclusion Criteria

We did not include studies with these characteristics:

- Studies based on animal models.

### 2.3. Information Source and Search Process

A systematic literature search and a critical appraisal of the collected studies were conducted. A bibliographic search using 3 databases (PubMed, Embase, Web of Science) has been carried out from the inception of these databases to 10 November 2021. The articles of interest were analyzed in full-text version and selected by an initial reviewer (G.D.); the results were compared with those of a second reviewer and the term (N.D.F), following an open discussion among all of the authors (N.D.F., G.D., P.F., and V.F.). Articles that respected the inclusion criteria were admitted. Only papers or abstracts in English were included in the search. No unpublished or grey literature was searched.

The search for relevant articles on electronic databases was carried out using the following search criteria: “Deep venous thrombosis” OR “Deep vein thrombus” OR “Pulmonary embolism” OR “Thrombus” AND “Histological age” OR “Age determination” AND “Forensic pathology” AND “Immunohistochemistry” in the title, abstract, and keywords. The outcome chosen for this systematic review is the “thrombus age determination” as well as the validation of the immunohistochemical techniques currently available for this purpose.

## 3. Results

### 3.1. Study Selection

**Figure 1:**
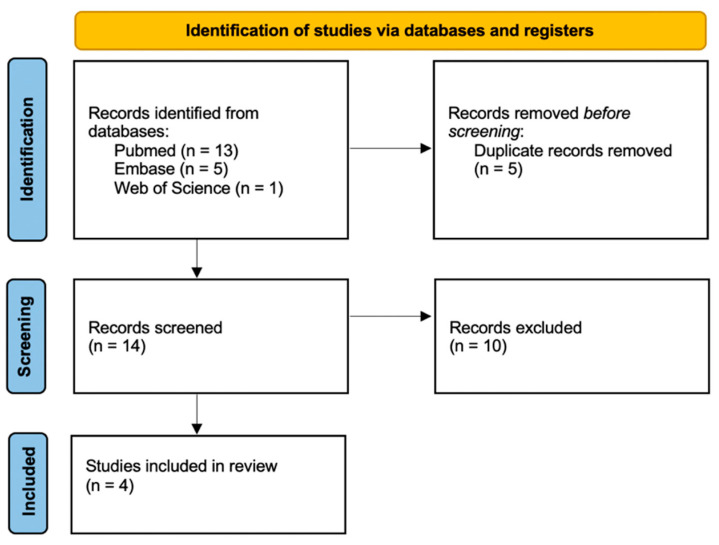
PRISMA 2020 flowchart showing our working methodology [32].

The first database-based search produced a total of 19 articles, of which 5 were duplicates. After excluding duplicate items from the selection, 14 articles were reviewed through title and abstract. Subsequently, 10 articles [33,34,35,36,37,38,39,40,41,42] were excluded because they did not meet the inclusion criteria (studies based on experimental animal models). Finally, 4 articles were included for qualitative synthesis (Figure 1) [28,30,43,44].

### 3.2. Study Characteristics

The included studies were performed in Italy and Japan. The articles were published in English between 2009 and 2019. From the point of view of the type of study, one article was a case report [43] and three articles were retrospective studies based on the analysis of samples previously collected [28,30,44]. A total of 157 DVT and 171 PTE samples were taken. From the point of view of the population considered, the 187 total subjects consisted of 90 males 97 females, with an age between 35 and 87 years. Thrombotic pathology was found in all cases of the studies considered, while PTE was found only in the autopsy findings related to three studies [30,43,44].

Concerning the immunohistochemical analysis techniques used, DVT samples were subjected to a reaction with anti-myeloperoxidase (anti-MPO) antibodies (1 case), fibrin, integrin α2bβ3, CD163, SMA, glycophorin A and CD206 (16 cases), CD34 (17 cases), anti-fibrinogen, CD15, CD45 and CD61 (140 cases), and CD68 (157 cases). Instead, PTE samples were treated with anti-CD34, anti-MPO (1 case), CD3 (30 cases), fibrinogen, CD15, CD61 (140 cases), CD45 (170 cases), and CD68 (171 cases). A schematic model of the techniques used by the articles considered is available in Table 1.

### 3.3. Risk of Bias

The merits of this review consist in the soundness of the sources drawn, in the breadth of the studies involved, in the rigorous methodology used for research, and the selection of sources (as visible in the relative flow diagram). It is worth noting that the 11-year time interval between the studies, despite our maximum commitment to standardized evaluation of the results, makes it essential to use a clinical filter in the interpretation of the results, especially in a rapidly evolving field of research such as the one under consideration. An overview of the main heterogeneities of the studies examined can be found in Table 2.

### 3.4. Main Findings from Reviewed Articles

The inclusion criteria adopted for the selection of studies have been illustrated in the previous paragraphs. However, the importance and the scientific weight of these have not been properly highlighted yet. The first step on the field has been taken by Fineschi et al. [30], who associated immunohistochemical analysis with traditional histological methods, discovering a considerably positive correlation between the two methods.

After a few years, Furukoji et al. [28] conducted further post-mortem studies on human subjects. This experience allowed for the discovery of new suitable markers on DVT timing perspective.

Moreover, scientific panorama offered the single case study of Maffeis et al. [43], who applied the original methodologies proposed by Fineschi [30] and Nosaka [33] to a clinical case considered as a whole (DVT and PE samples from the same subject).

Furthermore, the greatest contribution made by Mansueto et al. [44] has been the proposal of a new “Inflammatory infiltration and fibrosis score” to be applied to a totally new field, consisting of a strict five phases distinction of the early 72 h from thrombosis onset.

## 4. Discussion

The histological analysis of biological samples is a procedure that, over the years, has allowed us to obtain amazing results based on the use of several reactions, as well as thanks to the increasing knowledge about the underlying physiopathological processes. Thus, many authors, using techniques that have been available for decades or even centuries, proposed several models of age determination that rely on the most common microscopic findings [30,31,45,46,47,48,49].

Several authors have tried to determine the age of thromboembolism by dividing chronological phenomena into phases. Moller [45] made the first attempt to subdivide the morphological changes of the thrombi for forensic use in 1923. Later in 1977, another attempt was made by Sevitt [46], but the basis for the chronological staging of thromboembolism is due to the results of experiments conducted by Irninger [31] in 1963.

**Figure 2:**
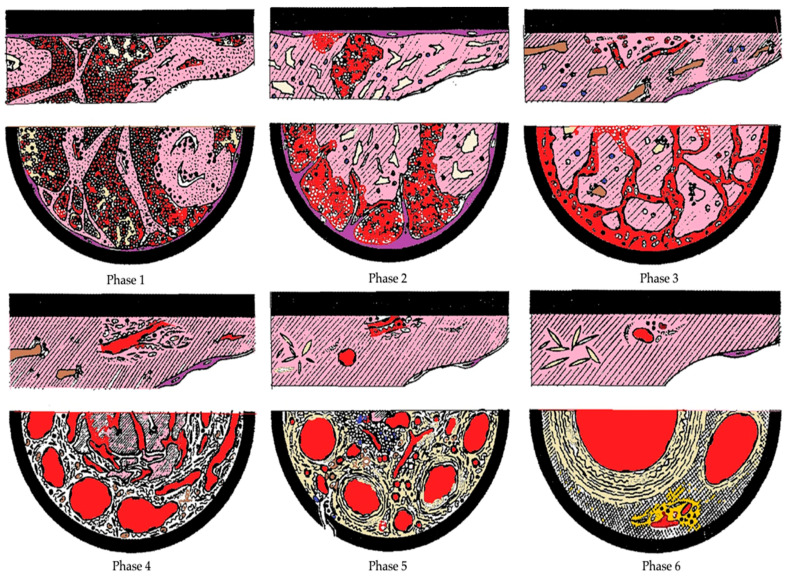
Images of the chronology of the microscopic changes related to the organization of the thrombus. Phase 1: Absence of reaction between endothelium and thrombus; leukocytes, platelets, and fibrin streaks are unaffected; erythrocytes agglomerated centrally and scattered peripherally. Phase 2: At day five, penetration of endothelial buds; initial hyalinization, mainly central; pycnotic leukocytes and mononuclear cells enlarged; thrombus contraction may create fissures and cavities with erythrocytes inside. Phase 3: By day 10, first capillaries, fibroblasts, mesenchymal cells and histiocytes with accumulations of hemosiderin; thrombus hyalinized and divided into large clumps; residual leukocyte nuclei. Phase 4: From week four, argyrophilic fibers and collagen; numerous capillaries. Phase 5: From the eighth week to the eighth month, completely hyalinized thrombus and presence of fusiform cholesterol crystals; vascularized loose connective tissue; centrally sinuous spaces traversable by fresh blood. Phase 6: After the sixth month, almost complete recanalization through large vessels separated by compact, fibrous connective tissue poor in cellular elements (modified from Irninger W. et al. [31]).

Subsequent contributions to the histological age determination of the thrombus were given by the experiences of Olsen [47] and Leu [48], thanks to studies conducted in the 1980s. These studies, overall, have allowed the histological transformation of thrombi to be divided into 6 phases, analyzing an overall period of about 12 months (Figure 2).

Despite the accuracy of such a chronological distinction, authoritative authors such as Janssen [49] and Knight [29] express a substantial warning of caution in using an excessively detailed scheme for use in the forensic setting.

Therefore, to date, the international staging of reference is that proposed by Fineschi [30], according to which, in the forensic setting only three phases can be distinguished with a degree of certainty (Table 3).

Following the latter model, in phase 1, between the 1st and 7th day, platelet plugs associated with fibrin deposition are formed, with a layer growth (lines of Zanh) in the absence of endothelial reaction. The integrity of the red blood cells is associated with early white blood cell pyknosis and von Kossa’s reaction shows calcium precipitation. Monocytes show enlarged nuclei. The differential diagnosis between thrombus and a clot is made possible both by analyzing the different relationships they contract with the endothelium and by analyzing their different physical composition. While in the early stages the clot is firmly attached to the endothelium and is not easily removable without leaving fragments in situ, a clot is not attached to the endothelium and can be easily removed. Secondly, while the thrombus undergoes the modifications of the physical composition that we have previously illustrated, a clot maintains its blood composition: mainly red blood cells, leukocytes and platelets, and a fibrin network.

In phase 2, identified up to 8 weeks, there are organizational phenomena of endothelial gemmation and penetration of fibroblasts with consequent proliferation within the medial ring, siderophages, coalescence of the fibrinous net, and endothelial proliferation on the surface of thrombus.

**Figure 3:**
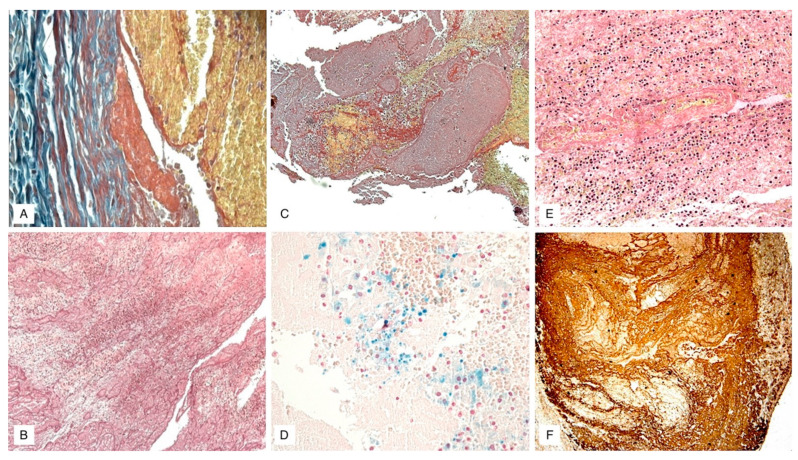
Images of the chronology of the microscopic changes related to the organization of the thrombus, according to Table 3. ((**A**,**B**) Phase 1, Mallory trichrome and phosphotungstic acid-hematoxylin (PTAH) staining; 100×, 60×. (**C**,**D**) Phase 2, Van Gieson and Perls’ iron staining; 60×, 100×. (**E**,**F**) Phase 3, Van Gieson and Weigert–Van Gieson staining; 80×, 60×) [30]. All images refer to samples of thrombi from deep veins of lower limbs of human patients.

Finally, phase 3, which includes thrombus older than two months, sees a complete hyalinization of the thrombus with sinuous central cavities, with poor cell representation and recanalization to blood flow with large blood-containing neo-vessels [30] (Figure 3).

On the basis, therefore, of the knowledge acquired about the histological modifications that a clot or embolus encounters during its organization and recanalization, several studies used different markers to highlight the various and different cells and structures that make up the thrombus and that succeed in its transformation. The various authors testing the positivity or not at these markers aim to place the examined thrombus sample in one of the phases described above and then determine the time of onset.

The important progress in the immunological field has involved the possibility of adopting new techniques for microscopic analysis based on an antigen–antibody reaction. This resulted in the development of immunohistochemistry. Many authors, as a result, engaged in research of thrombo-specific antigens that could increase the sensitivity and diagnostic specificity of microscopy in the field of DVT and PTE, thus contributing to the current knowledge about thrombus age determination [30,33,34,35,36,37,38,39,40,41,42,50,51]. Because of the lack of a scientific study that was comprehensive of such research, the compilation of the present systematic review was necessary, aiming to highlight the current state of the art in the matter.

Firstly, it is remarkable that the scientific evidence obtained so far seems to agree with the traditional histological model which is currently internationally adopted [30]. Because the multitude of animal model studies currently available still need validation on the human model, we have only analyzed the data from human studies.

The initial starting point of this group of research is represented by the study of Quarmby et al. [50], who in 1999, used a model of ligature of the saphenous vein in subjects undergoing surgical procedures, in order to induce in a controlled manner, the formation of thrombus then subjected to immunohistochemical investigations. The iatrogenic induction of the thrombotic process by intravascular administration of autologous thrombin did not allow for obtaining totally spontaneous samples. However, this study revealed a potential utility for dating early-stage thrombosis of P-selectin and VCAM-1 adhesion molecules.

Subsequently, Modarai et al. [51], based on previous studies on animal models, observed an important link between the activity of monocytes/macrophages (CD68+) and thrombus recanalization processes, following the production of pro-angiogenic factors. More recently, Nosaka has studied, through the use of immunohistochemical techniques, a model of stasis-induced DVT in mouse models: specifically, a 2009 study [33] compared the level of neutrophils (MPO+) and macrophages in clots of different ages, to establish a link between the time elapsed since the formation of thrombus and an inversion of the ratio between neutrophils and macrophages (N/M ratio) (Table 4).

Further research [35] also conducted on animal models allowed for establishing how an increase in fibroblasts (SMA+), involved in revascularization processes, was shown in samples starting from 7 days, with evident preponderance starting from 10 days. Their results are consistent with those of previous human studies, although they do not allow for definitive confirmation.

The fervent activity in the field of animal experimentation, despite the brilliant results achieved, still needs confirmation on human models; the examination of these perspectives, therefore, needs to be carried out separately.

### Immunohistochemical Findings

Despite the significance of the investigations mentioned above, the first real contribution to the age determination of human thrombi/emboli, taken from human corpses in the field of forensic pathology, was provided by Fineschi et al. [30] through the application of suitable histochemical markers which were compared with the histological model described above. Fineschi et al. adopted, therefore, antibodies anti-fibrinogen, anti-platelet (CD61), anti-leucocytes (CD45), anti-neutrophils (CD15), and anti-macrophages (CD68). In particular, comparing the immunohistochemical samples with the histological staging proposed by Fineschi himself, the authors analyzed three phases of modification of the thrombus [30,31,32,33,34,35,36,37,38,39,40,41,42,43,44,45,46,47,48,49,50,51,52].

Phase 1, comprising the first seven days of onset, showed a marked positivity for the platelet CD61 antigen in the absence of an endothelial reaction; the leukocytes, evidenced by CD15, CD45, and CD68 antigens, showed signs of pyknosis and nuclei enlargement.

Phase 2, instead, between the second and the eighth week, showed signs of organization of fibrin, evidenced by anti-fibrinogen antibodies, with entrapment of neutrophils CD15; there was also a marked increase in the number of macrophages (CD68), an aggregation of platelets, highlighted by the anti-CD61 antibody, and the presence of scattered nuclear debris of white blood cells (CD45).

Finally, phase 3, starting from the third month, was characterized by the presence of a compact connective tissue, hyaline, fiber-rich, and for the poor representation of the leukocyte population (CD45 and CD68). A key finding from this research is the correlation between the histologically detected phases by hematoxylin-eosin (EE), trichromic and von Kossa stains, and the immunohistochemical investigation data: in other words, the actual applicability of this technique to the intended purpose was demonstrated. Therefore, the authors of the study concluded by stating the objective usefulness of immunohistochemical dating of thrombus/embolus, associating this technique with traditional histology to more accurately identify morphological, structural, and numerical cells modifications involved in such processes.

Subsequently, Furukoji et al. [28] introduced an immunohistochemical analysis to date DVT aspirates in living subjects, in the absence of pulmonary embolic complications. Immunological markers used in this work allowed to highlight fibrin aggregates, platelets (integrin α2bβ3), neo-vessels (α-smooth muscle actin or SMA, CD34), erythrocytes (glycophorin A), and macrophages (CD68, CD163, and CD206). The analysis of the results obtained, carried out by the Spearman’s rank correlation coefficient, showed interesting results: the number of erythrocytes (glycophorin A) seemed to negatively correlate with the time of onset (correlation coefficient: −0.76; *p*-value: 0.001), thus giving to this marker an important identifying role of the acute phase; the macrophages CD68 and CD163-positive seemed instead positively correlated with the time of onset (respectively, correlation coefficient: 0.60 and 0.64; *p*-value: 0.05 and 0.01).

The latter, in particular, because of their hemophagocytic role [51] and stimulating the growth of mesenchymal stem cells, were highly represented in the erythrocytic population, SMA, and CD34 cells reactive areas [52]. This, therefore, makes it possible to confirm macrophages as a marker of the organizational phase of the clot. Finally, the poor positivity of the CD206 marker has been attributed to the low importance that this macrophagocytic clone has in the field of DVT, being more characteristic of the human atherosclerotic lesions with intraplaque hemorrhages [53].

There was no significant correlation in this study between revascularization markers and the age of thrombus (SMA and CD34). However, this result is not a final verdict, as it is very likely due to a bias that is inherent to the method of sampling adopted in the study. The vascular wall was absent in combination with the sample of thrombus, thus making the latter only a portion of the proper sample.

The case report of Maffeis et al. [43], despite the poor heterogeneity of the study, has been included in this systematic review as it is characterized by the completeness of the clinical picture (DVT and PE) and because it was performed in full compliance with the isolation and evaluation protocols of histological/immunohistochemical samples proposed by Fineschi [30] and Nosaka [33]. Samples of the left femoral vein, lung tissue, and right ventricle of the heart have been subjected to histological staining and immunohistochemical investigation through the use of anti-CD34 antibodies (neo-vessels), CD68 (macrophages), and MPO (neutrophils). This single study clearly showed the importance of comparing the histological and immunohistochemical results for accurate process dating: the DVT sample, showing positive for CD34, showed endothelial budding phenomena; the N/M ratio derived from the positivity for the markers MPO and CD68 was <1. The free surface of the thrombus and the absence of phenomena of recanalization of the same allowed therefore to place temporally the thrombus inside phase 2 according to the classification by Fineschi et al. [30].

Lung samples, on the other hand, despite a discrepancy between the N/M ratio of the pulmonary trunk (1) and the minor pulmonary vessels (>2), showed no reaction to the CD34 marker and poor presence of hemorrhagic deposits to the Perls’ stain. This allowed attributing to these samples more recent dating, traceable to phase 1 by Fineschi et al. [30].

Finally, the study by Mansueto et al. [44] allowed the immunohistochemical investigation to be applied to PE samples of 30 subjects using leukocyte (CD45), T/monocyte lymphocytes (CD3), and macrophages-(CD68) specific markers.

Further analyses were conducted by immunofluorescence using anti-factor VIII and fibrinogen antibodies. Histochemical Sirius Red/Fast Green staining was also performed for the observation of collagen. The absolute novelty of this study consists in the attempt to establish even narrower time limits for the fatal EP age determination, through the use of a 3-point inflammatory cellular infiltration and fibrosis semi-quantitative score (Table 5).

The application of this score to the thrombus age determination has allowed, therefore, for the construction of a 5-phase algorithm (Table 6).

The first phase (covering the first 60 min) does not show lymphocyte infiltration (CD3) in the immunohistochemistry test but showed an intense accumulation of platelets in poor association with erythrocytes thanks to immunofluorescence. The second phase (within the first 24 h) shows a small positivity of CD3 inside the thrombus. The third phase (between 24 and 48 h) allows instead for appreciation an increase of the cell infiltrate, which is meanwhile associated with an initial cellular degradation at the periphery of the thrombus with the recruitment of macrophages and fibroblasts.

The lymphocyte infiltration and fibrosis increase characterize the fourth phase (between 48 and 72 h). In the fifth and final phase, after 72 h, it was possible to observe endothelial cell proliferation and initial thrombus recanalization. Despite these brilliant results, the authors conclude this study stating that there is a very high risk of errors during immunohistochemical investigations on cadaver samples, for reasons related also to the loss of antigenicity by post-mortem tissues. Therefore, the authors recommended performing a histological approach first. The latter might be followed by immunohistochemical techniques in order to increase its sensitivity.

## 5. Conclusions

Concerning the three essential phases of the organization of the thrombus previously postulated by Fineschi et al. [30], we can affirm that the use of specific immunohistochemical markers allows for increasing the investigation’s diagnostic accuracy [54].

Effective identification of the first phase of the process (within the seventh day) is allowed by: the detection of platelet activity through CD61 antigen, the evidence of the integrity of erythrocytic elements presenting glycophorin A antigen, and the initial evidence of leukocyte populations using CD15 antigens, CD45, and CD61.

Both for the first and the second phases, however, an initial scientific consensus on the applicability of the N/M ratio is reached. The latter was proposed by Nosaka et al. [33] and is based on the relationship between MPO+ and CD68+ populations.

This algorithm, however, still needs a large-scale application to validate its reliability and its reference values. The Mansueto proposal [44] appears even more interesting, as it consists of a score that combines the level of lymphocytic invasion and thrombus fibrosis. This score aims to further subdivide the first phase into five subgroups. The earlier of those subgroups would allow for identifying typical processes that occur within the first 60 min from the formation of the thrombus/embolus.

Such a proposal, despite the risk inherent in such a sensitive model, in light of the wide time ranges currently used in the international arena, appears to be supported by solid scientific evidence.

Therefore, as previously stated, further extensive studies are required to obtain international scientific validation.

Concerning the classification of the second phase, the current scientific proposal consists in quantitatively evaluating the growing leukocyte population using the N/M ratio. In addition, this proposal consists in highlighting the thrombus organization using anti-fibrinogen and anti-fibrin antibodies, as well as the SMA antigen. The latter would allow us to highlight endothelial budding and channeling processes.

In addition, the leucocyte antigens mentioned above would, according to quantitative principles, effectively establish the age determination of thrombus even in later stages, permeating the histological evidence of the thrombus hyalinization.

In conclusion, among the most important conclusions drawn from the analyzed studies, it is worth emphasizing the importance of taking samples as complete as possible [55]. The latter should also include the vascular wall. The presence of the vascular wall within the sample would allow for evidence of the continuous mutation of the relationship between vascular endothelium and thrombus. On the other hand, in the absence of peri-lesional endothelium, the sampling should be considered incomplete and unreliable for age determinations purposes [56].

Finally, the need for close integration between histological, histochemistry, immunohistochemistry, and immunofluorescent techniques finds full agreement between the authors.

To date, it is not possible to determine whether immunohistochemistry will develop to the point of becoming independent from traditional methods. The present state of the art, which finds agreement in the available international literature, provides that antigen–antibody reaction should be carried out after a first histological staging, to further reduce the time window and, thus, improving the accuracy of age determination. Such an antibody–antigen reaction should also be carried out in case a sample cannot be assessed as to whether the sample should place a finding in one phase or another with certainty.

Immunohistochemistry can be used when doubtful findings do not allow for classifying samples within one phase or the other using traditional histology.

This contribution to the international scientific community is therefore a warning not to use only immunohistochemistry, but above all, it represents an invitation to its auxiliary use to allow for its further development, finally leading to the validation of principles hitherto limited to animal application.

## Figures and Tables

**Table 1:** Summary of antibodies used in the studies considered and type and number of samples.

Used Antibodies	Studies Included in Systematic Review			
	Fineschi et al. 2009 [30]	Furukoji et al. 2016 [28]	Maffeis et al. 2017 [43]	Mansueto et al. 2019 [44]
Fibrin		16 (DVT)		
Fibrinogen	140 (DVT + PTE)			30 (PTE)
MPO			1 (DVT + PTE)	
Integrin α2bβ3		16 (DVT)	1 (DVT + PTE)	
SMA		16 (DVT)		
Glycophorin A		16 (DVT)		
CD3				30 (PTE)
CD15	140 (DVT + PTE)			
CD34		16 (DVT)	1 (DVT + PTE)	
CD45	140 (DVT + PTE)			30 (PTE)
CD61	140 (DVT + PTE)			
CD68	140 (DVT + PTE)	16 (DVT)	1 (DVT + PTE)	30 (PTE)
CD163		16 (DVT)		
CD206		16 (DVT)		
Factor VIII				30 (PTE)

**Table 2:** Bias assessment between selected studies.

Heterogeneities	Studies Included in Systematic Review			
	Fineschi et al. [30]	Furukoji et al. [28]	Maffeis et al. [43]	Mansueto et al. [44]
Year	2009	2016	2017	2019
Country	Italy	Japan	Italy	Italy
Type of Study	Retrospective Study	Retrospective Study	Case Report	Retrospective Study
Patient Selection	Post-Mortem Examination of Fatal PTE Cases	Alive Clinical-Diagnosed DVT Patients	Fatal PTE Case	Post-Mortem Examination of Fatal PTE Cases
Number of Patients	140	16	1	30
Patients Sex	63 Males 77 Females	8 Males 8 Females	1 Female	19 Males 11 Females
Age Range	37–73 Years	35–78 Years	46 Years	38–87 Years
Specimen Typology	DVT and PTE	DVT	DVT and PTE	PTE
Vessel Wall Collection	Yes	No	Yes	Yes
Histological Comparison	Yes	Yes	Yes	Yes
Histological Techniques	H&E, Masson, Azan, Mallory, PTAH, Van Gieson, Perls, von Kossa	H&E	H&E, Van Gieson, Perls	H&E, Picro Sirius Red/Fast Green
Other Employed Techniques	Confocal Laser Scanning Microscope (CLSM)	-	-	Immunofluorescence

**Table 3:** Histological age determination of thromboses and embolisms according to Fineschi et al. (2009) [30].

Phase	Histological Modification
1st Phase (1–7 Days)	Flowing blood on an eroded endothelium, eliciting a platelet plug and fibrin deposition with a layered growth (Zahn’s lines). No reaction between endothelium and thrombus is visible. Erythrocytes are preserved and agglomerated. Initial white blood cells pyknosis. Monocytes cells with enlarged nuclei. Calcium is observed as precipitates with von Kossa stain. The thrombus at its initiation is firmly attached to a small portion of the vessel wall and is not easily removed to leave fragments in situ. On the contrary, a coagulum maintains the usual blood composition (i.e., prevailing red cells plus leukocytes and platelets and a fine network of fibrin), is not attached to the endothelium, and can be easily removed.
2nd Phase (2–8 Weeks)	Endothelial budding and proliferative changes of the medial ring are represented by the penetration of fibroblasts. Macrophages containing hemosiderin predominate, red blood cells ghosts and fibrinous transformation. The ribbons of fibrin changing to coalescences, trapping white cells. The free surface of thrombus is covered by the endothelium. Scattered nuclear debris of white blood cells
3rd Phase (More than 2 Months)	Completely hyalinized thrombus with central sinuous cavities and more advanced recanalizing neo-formed larger vessels with fresh flowing blood. Few white cells are visible between compact, fiber-rich and cell-deficient connective tissue.

**Table 4:** DVT timing according to N/M ratio.

	Time Elapsed since the Formation of Thrombus (Days)		
	1 Day	1–3 Days	5 Days or More
N/M Ratio *	>5 (6.8 ± 1.1)	>2 (2.5 ± 0.4)	> or = 1 (1.1 ± 0.1)

* ratio between neutrophils and macrophages.

**Table 5:** Histological score of inflammatory infiltrate and fibrosis according to Mansueto et al.

Histological Findings	3-Point Inflammatory Cellular Infiltration and Fibrosis Semi-Quantitative Score			
	0	1	2	3
Cellular Infiltration	Absent	Little	Moderate	High
Fibrosis	Absent	10–40%	40–80%	>80%

**Table 6:** Score related to dating thrombus formation according to Mansueto et al.

	Early (1 h)	Recent (1 h–24 h)	Recent-Medium (24 h–48 h)	Medium (48 h–72 h)	Old (>72 h)
Inflammatory Cells	0	1	1/2	2/3	0/1
Fibrosis	0	0	1	2	3

## Data Availability

Not applicable.
